# Male-Dominant Spinal Microglia Contribute to Neuropathic Pain by Producing CC-Chemokine Ligand 4 Following Peripheral Nerve Injury

**DOI:** 10.3390/cells14070484

**Published:** 2025-03-23

**Authors:** Fumihiro Saika, Tetsuya Sato, Takeru Nakabayashi, Yohji Fukazawa, Shinjiro Hino, Kentaro Suzuki, Norikazu Kiguchi

**Affiliations:** 1 Department of Physiological Sciences, School of Pharmaceutical Sciences, Wakayama Medical University, Wakayama 640-8156, Japan; f-saika@tumh.ac.jp; 2 Faculty of Wakayama Health Care Sciences, Takarazuka University of Medical and Health Care, Wakayama 640-8392, Japan; 3 H.U. Group Research Institute G.K., Tokyo 197-0833, Japan; takeru.nakabayashi@hugp.com; 4 Department of Anatomy, Kansai University of Health Sciences, Osaka 590-0482, Japan; fukazawa@kansai.ac.jp; 5 Department of Medical Cell Biology, Institute of Molecular Embryology and Genetics, Kumamoto University, Kumamoto 860-0811, Japan; s-hino@kumamoto-u.ac.jp; 6 Faculty of Life and Environmental Sciences, University of Yamanashi, Yamanashi 400-8510, Japan; k-suzuki@yamanashi.ac.jp

**Keywords:** allodynia, CCL4, CCR5, female, inflammation, sex, spinal cord

## Abstract

Recent studies have revealed marked sex differences in pathophysiological roles of spinal microglia in neuropathic pain, with microglia contributing to pain exacerbation exclusively in males. However, the characteristics of pain-enhancing microglia, which are more prominent in males, remain poorly understood. Here, we reanalyzed a previously published single-cell RNA sequencing dataset and identified a microglial subpopulation that significantly increases in the spinal dorsal horn (SDH) of male mice following peripheral nerve injury. CC-chemokine ligand 4 (CCL4) was highly expressed in this subpopulation and its mRNA levels were increased in the SDH after partial sciatic nerve ligation (PSL) only in male mice. Notably, CCL4 expression was reduced in male mice following microglial depletion, indicating that microglia are the primary source of CCL4. Intrathecal administration of maraviroc, an inhibitor of the CCL4–CC-chemokine receptor 5 (CCR5) signaling pathway, after PSL, significantly suppressed mechanical allodynia only in male mice. Furthermore, intrathecal administration of CCL4 induced mechanical allodynia in both sexes, accompanied by increased expression of c-fos, a neuronal excitation marker, in the SDH. These findings highlight a sex-biased difference in the gene expression profile of spinal microglia following peripheral nerve injury, with elevated CCL4 expression in male mice potentially contributing to pain exacerbation.

## 1. Introduction

Pain serves as an essential manifestation of abnormalities in the body and helps in initiating protective actions [1,2]. However, pain exceeding the normal physiological range requires medical evaluation and intervention. Neuropathic pain, resulting from nervous system damage, often does not respond to conventional analgesics, and has prompted extensive research into the underlying molecular mechanisms [3,4]. Recent studies have highlighted activation of microglia in the spinal dorsal horn (SDH) and the role of pain-related inflammatory mediators, such as cytokines and chemokines, in exacerbating and prolonging the pain [5,6]. Emerging evidence is also revealing sex-based differences in the involvement of microglia in neuropathic pain [7,8]. We and other research groups have demonstrated that administering microglial inhibitors (e.g., minocycline and pexidartinib) to rodent models of neuropathic pain suppresses pain in males, but not in females [9,10,11]. These findings suggest that, in males, microglia play a direct role in pain exacerbation under pathological conditions and contribute to neuropathic pain. Conversely, this mechanism does not appear to operate in microglia of females, implying the involvement of alternative pathways in them.

Microglia express receptors that respond to various stimuli from their environment and neighboring cells, and their activation has been implicated in pain induction [12,13]. Several recent studies have revealed significant sex-based differences in this process. For example, intrathecal (i.t.) administration of lipopolysaccharide, a toll-like receptor 4 agonist, or of colony-stimulating factor 1 (CSF1), which is essential for the survival and proliferation of microglia, induces morphological activation of microglia and reduces the pain threshold in rodent models [14,15,16]. This effect is pronounced in male mice but absent in female mice. Additionally, we demonstrated that selective activation of spinal microglia using Gq-DREADD (designer receptors exclusively activated by designer drugs) induces pain in male mice, but not in female mice [17]. These findings underscore sex-dependent differences in spinal microglial responsiveness to specific stimuli and their involvement in pain under physiological conditions. Despite growing evidence gathered employing various tools used to manipulate microglial activity, the key molecules that define male-dominant microglial involvement in neuropathic pain have not been identified definitively. Identifying these molecules is essential for unraveling the sex-biased mechanisms of pain regulation.

Microglia typically produce inflammatory factors that act on surrounding cells [18,19]. Therefore, identifying the factors uniquely expressed in pain-enhancing microglia and characterizing these cells is critical. A previous study using single-cell RNA sequencing (scRNA-seq) identified unique subpopulations of spinal microglia predominantly arising in male mice following peripheral nerve injury [20]. Based on these findings, we reanalyzed spinal microglial transcriptomes at single-cell resolution to identify a key soluble factor, the chemokine CC-chemokine ligand 4 (CCL4), which is predominantly expressed in male microglia under pathological conditions. Furthermore, we validated the functional significance of CCL4 via conventional biochemical and behavioral assays and elucidated the mechanisms underlying sex differences in microglia-mediated neuropathic pain.

## 2. Materials and Methods

### 2.1. Mice

All animal experiments were approved by the Animal Research Committee of Wakayama Medical University and were conducted in accordance with the in-house guidelines for the care and use of laboratory animals at Wakayama Medical University, as well as the Animal Research: Reporting of In Vivo Experiments (ARRIVE) guidelines. Male and female C57BL/6 mice (6–8-weeks-old) were purchased from SLC (Hamamatsu, Japan) and used for the experiments at 8–12-weeks of age. All mice were housed in groups of 5–6 in plastic cages under controlled temperature (23–24 °C), relative humidity (60–70%), and a 12 h dark/light cycle, with free access to food and water.

### 2.2. Processing of scRNA-Seq Data

We analyzed scRNA-seq data from male and female mouse microglia using publicly available data from the gene expression omnibus data repository (accession number: GSE162807) [20]. This study included 142,905 cells collected before and after peripheral nerve injury (at 3 days, 14 days, and 5 months), which were aligned to the mouse mm10 reference transcriptome. To identify the cell types, we analyzed all microglia samples and integrated them using the Harmony method [21] in the Seurat single-cell analysis package (Version 5.1.0) [22]. Initially, cells expressing more than 4000 genes (potential doublets) or fewer than 500 genes, as well as those with more than 5% mitochondrial genes, were filtered out. After normalizing, selecting variable features, and scaling each dataset separately for the filtered 135,806 cells, we performed principal component analysis for dimensional reduction. We then used the *IntegrateLayers* function to combine all the sample datasets. The integrated dataset was processed using the *FindNeighbors* function (reduction = “harmony”, dims = 1:11) to obtain the nearest-neighbor graph, the *FindClusters* function (resolution = 0.3) to identify each cell population, and the *RunUMAP* function (reduction = “harmony”, dims = 1:11) for visualization of the integrated dataset using Uniform Manifold Approximation and Projection (UMAP) dimension reduction. This process resulted in the identification of 11 cell type clusters.

### 2.3. Gene Set Enrichment Analysis

To functionally annotate the marker genes in each cluster, we conducted gene set enrichment analysis (GSEA) [23] using the R package fgsea (version 1.32.0). First, we identified marker genes in each cell cluster in Seurat using the *FindMarkers* function with the parameters test.use = wilcox, min.pct = 0.01 and logfc.threshold = 0.1. For each cluster, we generated a gene list sorted in decreasing order of −log_10_ *p*-values and used this list for GSEA. After performing GSEA with the molecular signatures database (MSigDB) hallmark dataset [24], we considered gene sets enriched at an FDR < 0.05.

### 2.4. Partial Sciatic Nerve Ligation (PSL) Model

The mice were subjected to PSL, as previously described [25,26]. Briefly, under isoflurane anesthesia, the left common sciatic nerve of each mouse was exposed at the mid-thigh level by making a small skin incision on one side, hereafter referred to as the ipsilateral side. Approximately one-third of the sciatic nerve was tightly ligated with a silk suture (Natsume Seisakusho, Tokyo, Japan), followed by suturing of the muscle and skin layers and sterilization of the surgical area with povidone–iodine. The untreated right limb was considered the contralateral limb.

### 2.5. Administration of Pexidartinib (PLX3397)

To deplete macrophages and microglia in vivo, PLX3397 (MedChemExpress, Monmouth Junction, NJ, USA), a CSF1 receptor (CSF1R) inhibitor, was formulated into the AIN-76A rodent diet (Research Diets, New Brunswick, NJ, USA) at 290 mg/kg. The PLX3397 dose was established based on a previous report [27]. The mice had free access to the PLX3397-formulated diet for two weeks instead of normal food, as PLX3397 is orally active. The AIN-76A rodent diet was used as the control.

### 2.6. Immunohistochemistry

The lumbar (L4–5) spinal cord was harvested from euthanized mice following transcardial perfusion with phosphate-buffered saline (PBS) and fixed in 4% paraformaldehyde/phosphate-buffer solution. The specimens were post-fixed in 4% paraformaldehyde and cryoprotected in 30% sucrose/PBS solution at 4 °C overnight. After embedding in a freezing compound (Sakura, Tokyo, Japan), frozen tissues were longitudinally sectioned at 30 µm thickness using a cryostat (Leica Microsystems, Wetzlar, Germany), and the sections were floated in PBS. The sections were treated with PBS containing 0.1% Triton X-100 (PBST) for 1 h and then blocked with 5% donkey serum at room temperature (15–25 °C) for 2 h. The sections were then incubated overnight at 4 °C with primary antibodies targeting IBA1 (rabbit polyclonal, 1:1000; Fujifilm Wako, Osaka, Japan), NeuN (mouse monoclonal, 1:500; Millipore, Billerica, MA, USA), and c-fos (rabbit polyclonal, 1:50; Santa Cruz Biotechnology, Dallas, TX, USA). The sections were rinsed in PBST and incubated with fluorescent dye-conjugated secondary antibodies (1:200; Thermo Fisher Scientific, Waltham, MA, USA) at room temperature for 2 h. Finally, the sections were washed with PBS, mounted on glass slides, and covered with coverslips using the DAPI-Fluoromount-G (Southern Biotechnology Associates, Birmingham, AL, USA). Fluorescent images were acquired using a confocal laser-scanning microscope (Olympus, Tokyo, Japan). The number of IBA1^+^ cells within the lamina I-III of the SDH was measured in a square area (200 × 200 μm^2^) using the FLUOVIEW software (Version 2.5.1.228).

### 2.7. Reverse Transcription-Quantitative Polymerase Chain Reaction (RT-qPCR)

Mice were euthanized using isoflurane, and fresh dorsal horns of the lumbar (L4–5) SDH samples were collected in RNAlater solution (Thermo Fisher Scientific). Total RNA was isolated from tissues using the TRIzol Plus RNA Purification Kit (Thermo Fisher Scientific) following the manufacturer’s instructions. Briefly, tissues were placed in a 1.5 mL RNase-free tube and homogenized with TRIzol reagent. Chloroform was added to each sample, and the mixture was then centrifuged at 4 °C for 15 min. The aqueous phase containing RNA was transferred to a fresh tube, and RNA was isolated using a purification column. Total RNA extract (1 µg) was incubated with random primers (Promega, Madison, WI, USA) at 70 °C for 5 min and subsequently cooled on ice. Samples were converted into cDNA by incubation with M-MLV Reverse Transcriptase (Promega) and dNTP Mix (Promega). qPCR was performed using the AriaMx Real-Time PCR System (Agilent Technologies, Santa Clara, CA, USA) with template cDNA (10 ng), primers for each gene (Thermo Fisher Scientific), and SYBR Premix Ex Taq II (Takara Bio, Kusatsu, Japan). The reactions were performed under the following conditions: 3 min at 95 °C, followed by 45 cycles of step two comprising 10 s at 95 °C and 30 s at 60 °C. Fluorescence intensities were recorded and the data were normalized to β-actin expression (*Actb*). The primer sequences used were as follows: *Actb*, 5′-CAGCTGAGAGGGAAATCGTG-3′ and 5′-TCTCCAGGGAGGAAGAGGAT-3′; *Cd11b*, 5′-GTTTCTACTGTCCCCCAGCA-3′ and 5′-GTTGGAGCCGAACAAATAGC-3′; *Ccl4*, 5′-ATGAAGCTCTGCGTGTCTGC-3′ and 5′-GCCGGGAGGTGTAAGAGAAA-3′.

### 2.8. Drug Administration

A CC-chemokine receptor 5 (CCR5) antagonist (Maraviroc; Tocris Biosciences, Bristol, UK) was dissolved in dimethyl sulfoxide and diluted in sterile PBS for further use. Based on previous reports [9,28], 20 nmol maraviroc was administered intrathecally (i.t.) on day 7 after PSL. Recombinant CCL4 (BioLegend, San Diego, CA, USA) was dissolved in sterile PBS and i.t. administered at a dose of 1 or 10 pmol. Under isoflurane anesthesia, an i.t. injection was administered in the region between the spinal L5 and L6 vertebrae using a 30-gauge needle fitted with a Hamilton microsyringe [29].

### 2.9. Von Frey Test

The mechanical pain threshold was determined using the von Frey test, as previously described [17]. Briefly, mice were individually placed on a metal mesh grid floor (5 × 5 mm) and covered with an acrylic box. After a 2- to 3 h adaptation period, calibrated von Frey filaments (Neuroscience, Tokyo, Japan) were applied to the middle of the plantar surface of the hind paw through the mesh floor. The filament set used in this study consisted of nine calibrated von Frey filaments: 0.02, 0.04, 0.07, 0.16, 0.4, 0.6, 1.0, 1.4, and 2.0 g. Using the up–down method, testing began with the application of 0.4 g filament. Quick withdrawal, shaking, biting, or licking of the stimulated paw was considered a positive paw-withdrawal response. If no withdrawal response occurred, the next stronger stimulus was applied. Conversely, the next weaker stimulus was selected following paw withdrawal, in accordance with Chaplan’s procedure [30]. Once the response threshold was crossed (two responses were straddling the threshold), the 50% paw-withdrawal threshold was calculated based on these responses.

### 2.10. Gene Expression Analysis

Expression profiling of CCL4 (*Ccl4*) gene across a diverse range of normal tissues, organs, and cell lines in mice was visualized using BioGPS (http://biogps.org/).

### 2.11. Statistical Analysis

All data are presented as the mean ± standard error of the mean (SEM). To compare differences between two groups, a two-tailed Student’s *t*-test or Welch’s *t*-test was used. To compare the differences between the four groups with two factors, two-way ANOVA followed by Tukey’s multiple comparison test was used. Statistical analyses were performed using the GraphPad Prism software (GraphPad Software, Version 10.1.2, Boston, MA, USA), and statistical significance was set at *p* < 0.05.

## 3. Results

### 3.1. Expression of CCL4 by Male-Dominant Subpopulation of Spinal Microglia After Nerve Injury

**Figure 1:**
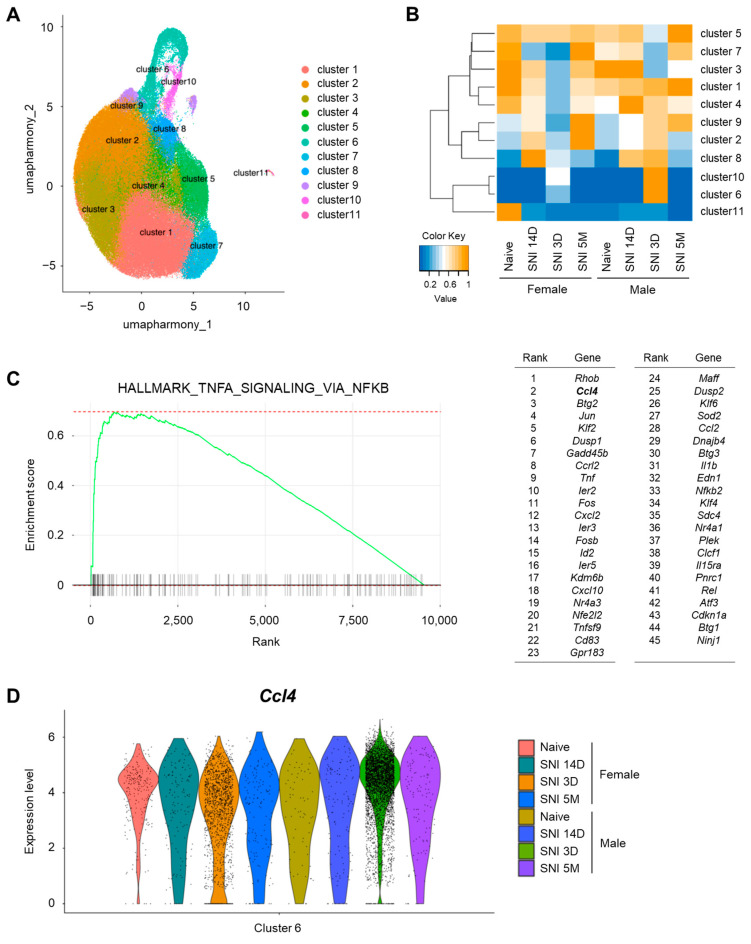
Male-dominant subpopulation of spinal microglia after nerve injury express CCL4. (**A**) Uniform manifold approximation and projection (UMAP) visualization of mouse microglia (*n* = 2 control samples and *n* = 6 injury samples from male and female mice), colored by cell type cluster. The control naive sample included 33,330 cells, whereas the spared nerve injury (SNI) model samples included 102,476 cells collected on day 3, day 14, and 5 months after SNI. (**B**) Heatmap based on cell number ratio. This heatmap indicates the ratio of cells classified into each cluster relative to the total number of cells in each sample. The vertical axis represents the cell type cluster numbers, whereas the horizontal axis represents the sample types. (**C**) Result of gene set enrichment analysis (GSEA) of marker genes in males compared with those in females for the “HALLMARK_TNFA_SIGNALING_VIA_NFKB” gene set. List of 45 leading edge genes are shown. (**D**) Violin plot for *Ccl4* genes, markers of cluster 6 cells.

To identify microglia-secreted pain-enhancing molecules in male-dominant subpopulations of activated microglia in the SDH after peripheral nerve injury (spared nerve injury: SNI), we reanalyzed a scRNA-seq dataset from a previously published study (Tansley et al.) [20]. Clustering analysis of microglia under the following conditions (day 3, day 14, 5 months after SNI and naïve controls for both sexes) revealed 11 distinct clusters (Figure 1A). Consistent with prior findings, canonical microglia genes, such as *Tmem119*, *Fcrls*, *P2ry12*, *Cx3cr1*, *Trem2*, and *C1qa*, were expressed in all cell type clusters, representing most of the analyzed microglia (Figure S1). In contrast, clusters 6 and 10, which accounted for a portion of the microglia, exhibited unique transcriptional profiles. The heatmap showed that the primary population in clusters 6 and 10 comprised microglia from male mice on day 3 after SNI, followed by female mice, whereas microglia from day 14 and 5 months after SNI, and naïve controls of both sexes were minimally represented (Figure 1B). Activated microglia play a crucial role in the development of neuropathic pain, highlighting the importance of early time points following nerve injury. Microglia categorized within clusters 6 and 10 showed an increase in both sexes on day 3 after SNI compared to naïve controls. However, the proportion of male microglia was greater than that of female microglia, suggesting that clusters 6 and 10 reflected sex-specific differences in activated microglia following nerve injury.

Given the pivotal role of inflammation-related molecules in the pathophysiology of neuropathic pain, we analyzed the gene expression patterns in these clusters according to sex. GSEA revealed activation of the TNFA_SIGNALING_VIA_NFKB pathway in cluster 6 microglia. Furthermore, 45 hallmark genes were identified as characteristic of male-dominant microglia after nerve injury. Although *Rhob* exhibited the most pronounced difference between males and females among all genes in cluster 6, we prioritized the soluble inflammatory molecule CCL4, which exhibited the second greatest sex difference. (Figure 1C). A violin plot for *Ccl4* expression levels across all microglia in cluster 6 confirmed that CCL4-expressing microglia were predominantly from male mice on day 3 after SNI (Figure 1D). Additionally, the number of CCL4-expressing microglia in males was greater than that in females in cluster 10 microglia (Figure S2), although the transcriptional profile of cluster 10 differed from that of cluster 6. These findings suggest that *Ccl4* expression is a defining feature of male-dominant microglia involved in neuropathic pain.

### 3.2. Male Microglia-Dominant Upregulation of CCL4 in the Spinal Dorsal Horn

**Figure 2:**
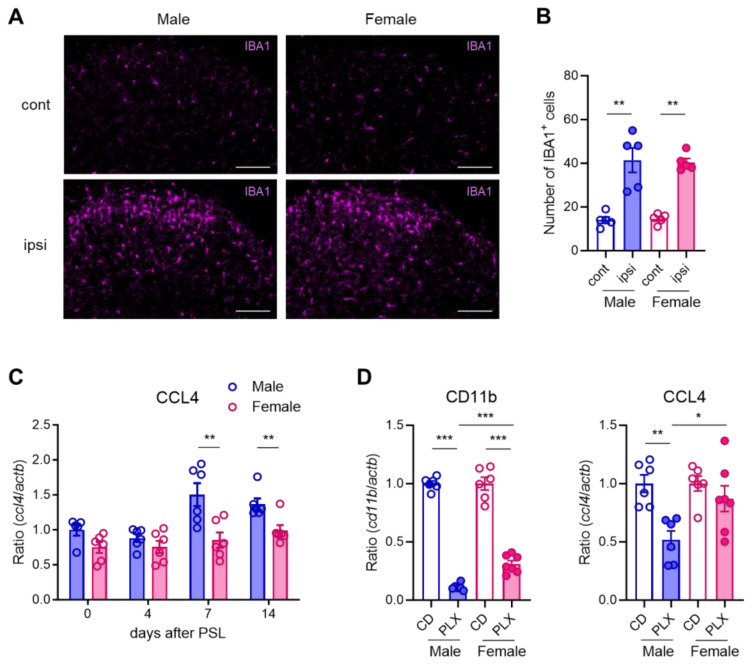
Male microglia-dominant upregulation of CCL4 in the spinal dorsal horn. Male and female mice were subjected to partial sciatic nerve ligation (PSL), and the lumbar spinal dorsal horn (SDH) was collected. (**A**) IBA1^+^ microglia in the SDH on day 7 after PSL were visualized using immunohistochemistry. Scale bars = 100 μm. (**B**) Quantitative analysis of the number of IBA1^+^ cells in the lamina I-III in the SDH (*n* = 5, Welch’s *t*-test, ** *p* < 0.01). (**C**) mRNA levels of *Ccl4* on days 0, 4, 7, and 14 after PSL were analyzed using RT-qPCR (*n* = 5–6, Student’s *t*-test, ** *p* < 0.01). (**D**) Mice were fed a control diet (CD) or PLX3397 diet for 7 days before PSL and subsequently subjected to PSL. The lumbar ipsilateral SDH was collected on day 7 after PSL. Fold changes in mRNA levels of *Cd11b* and *Ccl4* in PLX3397-fed mice compared with those in control-fed mice. (*n* = 6–7, Student’s *t*-test, *** *p* < 0.001, ** *p* < 0.01, * *p* < 0.05).

Using immunohistochemistry, we evaluated whether microglial activation differed in the SDH of male and female mice following PSL. On day 7 after PSL, the number of IBA1^+^ microglia was similarly increased on the ipsilateral side of the SDH in both male and female mice (Figure 2A,B). Next, we used RT-qPCR to assess the time course of *Ccl4* expression in the SDH after PSL. *Ccl4* was significantly upregulated on days 7 and 14 in male mice, but not in female mice, with expression levels being markedly higher in males at both time points (Figure 2C), whereas other pain-related genes, such as *Ccl3*, were similarly upregulated in both sexes [9]. As previously reported, treatment with PLX3397, an inhibitor of the CSF1 receptor, substantially reduced the expression of the microglial marker *Cd11b* in the SDH on day 7 after PSL in a sex-dependent manner. Consistently, *Ccl4* expression was significantly decreased upon PLX3397 treatment in male mice but remained unchanged in female mice (Figure 2D). A database search using BioGPS confirmed that *Ccl4* is expressed not only by peripheral macrophages but also by microglia (Figure S3). These findings indicate that *Ccl4* is upregulated in SDH microglia after PSL in males but not in females.

### 3.3. Sexually Dimorphic Effect of CCR5 Antagonist in Relieving Neuropathic Pain

**Figure 3:**
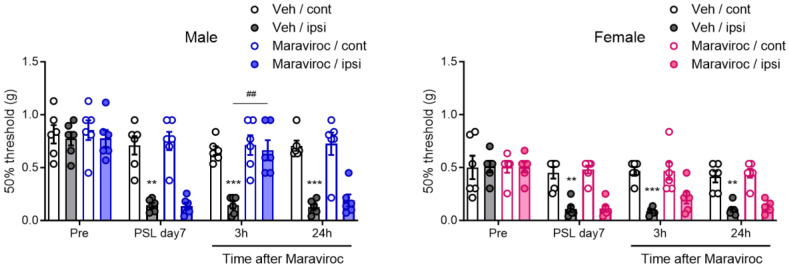
Sexually dimorphic effect of CCR5 antagonist maraviroc in relieving neuropathic pain. Male and female mice were subjected to PSL, and maraviroc (20 nmol) was intrathecally (i.t.) administered once on day 7 after PSL. The 50% mechanical threshold on days 0 (pre) and 7 after PSL, and 3 and 24 h after i.t. administration of maraviroc in male and female mice were assessed employing the up–down method using the von Frey test (*n* = 6, two-way ANOVA followed by Tukey’s multiple comparison test, *** *p* < 0.001, ** *p* < 0.01 vs. Veh/cont, ^##^ *p* < 0.01).

CCR5 is the principal receptor for CCL4, despite the complexity of the chemokine ligand–receptor system [31,32]. To assess whether the CCL4–CCR5 axis plays a sex-dependent pathophysiological role in neuropathic pain in the SDH, we investigated the effects of CCR5 blockade. Maraviroc, a CCR5 antagonist [33], was evaluated for its suppressive effects on PSL-induced neuropathic pain. Mechanical pain thresholds, assessed using the von Frey test, were significantly reduced on the ipsilateral side on day 7 after PSL in both male and female mice, confirming the development of mechanical allodynia. The i.t. administration of maraviroc (20 nmol) on day 7 transiently, but markedly, relieved PSL-induced mechanical allodynia 3 h after administration in male mice. In contrast, maraviroc did not suppress allodynia in female mice (Figure 3). These findings suggest that enhancement of the CCL4–CCR5 axis in the SDH plays a critical role in neuropathic pain in male mice.

### 3.4. Sex-Independent Allodynic Effects of CCL4 in the Spinal Dorsal Horn

**Figure 4:**
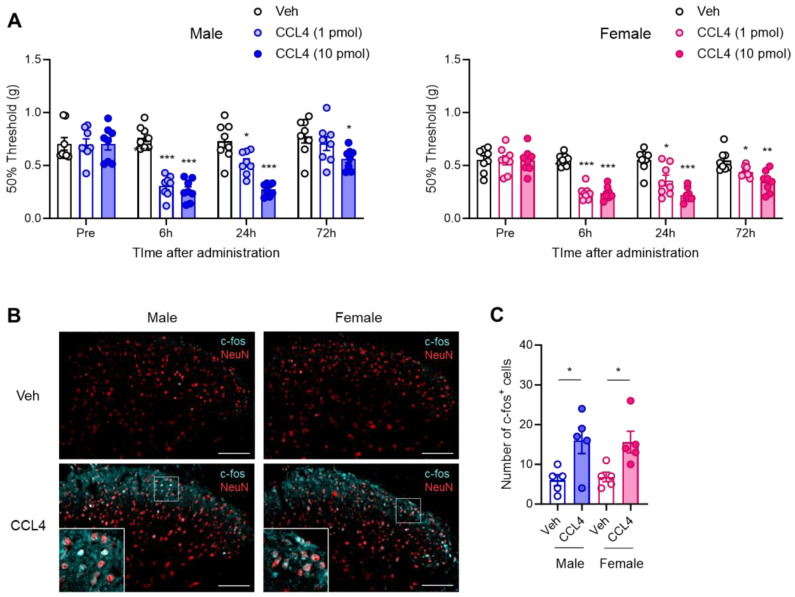
Sex-independent allodynic effects of intrathecally administered CCL4. Recombinant CCL4 (1 or 10 pmol) was intrathecally administered to naïve male and female mice. (**A**) The 50% mechanical threshold before and 6, 24, and 72 h after administration in male and female mice were assessed employing the up–down method using the von Frey test (*n* = 8, two-way ANOVA followed by Tukey’s multiple comparison test, *** *p* < 0.001, ** *p* < 0.01, * *p* < 0.05 vs. Veh). (**B**) Expression of c-fos protein in the SDH on day 1 after administration of CCL4 (10 pmol) was visualized via immunohistochemistry. Scale bars = 100 μm. (**C**) Quantitative analysis of the number of c-fos^+^ cells in the lamina I–III of the SDH (*n* = 5, Student’s *t*-test, * *p* < 0.05).

To determine whether an increase in CCL4 is sufficient to induce mechanical allodynia, we evaluated the effects of exogenous CCL4 on pain sensitivity in both male and female mice. A single i.t. administration of CCL4 (1 or 10 pmol) significantly reduced the mechanical pain threshold, indicating mechanical allodynia 6 h after administration in both sexes. The allodynic effects persisted for at least 72 h in a dose-dependent manner (Figure 4A). Further analysis showed that c-fos protein expression in the SDH was significantly increased in both male and female mice on day 1 after i.t. administration of CCL4 (10 pmol). These c-fos signals colocalized with NeuN, a marker of neuronal nuclei, indicating the activation of pain-processing neurons in the SDH (Figure 4B,C). These results indicate that CCL4 exerts a potent allodynic effect by reducing the mechanical pain thresholds in a sex-independent manner.

## 4. Discussion

In this study, we identified sex-biased characteristics of spinal microglia that contribute to the etiology of neuropathic pain. Bioinformatic analysis of published scRNA-seq datasets for spinal microglia revealed male-dominant expression of the chemokine *Ccl4* in specific microglial subpopulations following SNI. We further confirmed that *Ccl4* mRNA was upregulated in the SDH of males, but not females, after PSL, despite both sexes exhibiting similar morphological activation of spinal microglia. The i.t. administration of maraviroc, an inhibitor of the CCL4-CCR5 signaling pathway, significantly suppressed PSL-induced mechanical allodynia in male mice. However, the anti-allodynic effects of maraviroc were not significant in female mice. Notably, the i.t. administration of recombinant CCL4 induced mechanical allodynia in both sexes, indicating that the sex-dependent pathophysiological roles of microglia-derived CCL4 in neuropathic pain are mediated via its expression following peripheral nerve injury.

Following the discovery of a causal link between microglial activation and neuropathic pain [34], numerous studies have highlighted the roles of soluble factors derived from activated microglia in pain hypersensitivity. For example, inflammatory cytokines (e.g., interleukin-1β and tumor necrosis factor-α), chemokines (e.g., CCL2 and CCL3), growth factors, and lipid mediators have been shown to play critical roles in neuropathic pain by enhancing neuronal excitability and/or amplifying neuroinflammation [5,13,35,36]. This process is accompanied by the activation of microglia and astrocytes, ultimately leading to hyperexcitation of pain-processing pathways [6,37]. Although accumulating evidence suggests pronounced sexually dimorphic characteristics of microglia in the etiology of neuropathic pain [7,8,38], the existence of sex differences in the pathophysiological roles of these molecules remains underexplored. Elucidating the mechanisms underlying sex differences in microglia-driven neuropathic pain requires identification of critical molecules that define male-dominant microglial contributions to neuropathic pain.

In a previous study, Tansley et al. classified spinal microglia from naïve, sham, and SNI mice into 11 distinct clusters [20]. Among these, unique subpopulations (original clusters 7–9) were predominantly composed of microglia from SNI day 3 in both sexes. On SNI day 3, morphological activation and proliferation of microglia in the SDH were significantly increased in both sexes, and these microglia exhibited distinct transcriptome profiles compared to other subpopulations. Notably, the number of proliferative microglia in male mice was greater than that in female mice, suggesting sex differences in microglial characteristics [20]. We hypothesized that further analysis of their transcriptome data would help identify novel soluble molecules that drive functional sex differences in microglia. Our findings revealed that the TNFα signaling via the NF-κB pathway was upregulated in cluster 6, aligning with the established role of neuroinflammatory process in the etiology of neuropathic pain. Importantly, the proportion of microglia in male SNI day 3 was greater than that in female SNI day 3 within cluster 6, and CCL4 expressing microglia were primarily observed in males. Given that cluster 6 in our analysis aligns with previous findings [20], CCL4 emerges as a key molecule associated with sex differences in activated microglia after nerve injury.

Several neuropathic pain models have been established to investigate the etiology of neuropathic pain and to evaluate the therapeutic potential of drugs [39,40]. Among the four main nerve injury models, spinal microglial activation is a common feature, and the inhibition of activated microglia has been shown to alleviate neuropathic pain across these models [11,25,41,42]. However, the temporal dynamics of microglial activation and expression profiles of microglia-derived molecules may vary between models. To elucidate the common pathophysiological mechanisms underlying neuropathic pain, it is essential to examine key phenomena across different neuropathic pain models. Several studies have demonstrated significant microglial activation and upregulation of inflammatory molecules, sustained for at least two weeks after nerve injury, in neuropathic pain models [9,36,43]. Therefore, although Tansley et al. employed the SNI model [20], their scRNA-seq transcriptome data can be used to investigate the mechanisms underlying PSL-induced neuropathic pain. Indeed, the male-dominant upregulation of CCL4 after PSL in our study indicates the existence of shared mechanisms between the SNI and PSL models.

CCL4, also known as macrophage inflammatory protein-1β, exerts diverse effects on various cell types via its interaction with CCR5. Several lines of evidence suggest that the CCL4–CCR5 axis facilitates inflammation that underlies intractable diseases, such as osteoarthritis and diabetes, and promotes tumor development and progression by modulating lymphocytes, macrophages, and tissue-resident cells [44,45,46,47]. Additionally, CCL4 is upregulated under pathological neurogenic conditions following nerve injury, and inhibition of macrophage- or Schwann cell-derived CCL4 has been shown to attenuate neuropathic pain [48]. CCL4 also plays a crucial role in several neuroinflammatory diseases of the central nervous system, including traumatic brain injury and Alzheimer’s disease [47,49,50]. Thus, CCL4 upregulation likely contributes to pain hypersensitivity at the spinal level. A previous study reported that CCL4 was upregulated in the SDH following chronic constriction injury and that i.t. administration of maraviroc attenuated neuropathic pain in male rats [28]. However, the study did not address sex-related differences. In the present study, we demonstrated that CCL4 was upregulated exclusively in male mice, and that the CCL4–CCR5 signaling pathway was enhanced in the SDH following peripheral nerve injury in a sex-dependent manner.

Given that perineurally administered maraviroc at the site surrounding the injured sciatic nerve exhibited anti-allodynic effects in both male and female PSL models, as previously reported [9], it is surprising that i.t. administered maraviroc showed anti-allodynic effects exclusively in male PSL models. Despite the sexually dimorphic anti-allodynic effects of i.t. administered maraviroc, the i.t. administration of CCL4 induced robust allodynia in both sexes, whereas a previous report presented an allodynic effect of i.t. administered CCL4 only in males [51]. These findings suggest that activation of CCR5 in the SDH leads to pain hypersensitivity in both males and females. CCR5 is highly expressed in immune cells, including spinal microglia, and is also known to be expressed in neurons [51,52,53,54]. Indeed, the induction of c-fos expression following CCL4 administration supports the excitation of pain-processing neurons. Interestingly, CCL3, another CCR5 ligand, was similarly upregulated in the SDH of both sexes after nerve injury, as previously reported [9]. However, CCL4 upregulation showed clear sex differences, being elevated only in males. This transcriptional disparity may underlie sex-biased characteristics of microglia. Understanding the mechanisms underlying these sex-related differences is crucial. We previously demonstrated that the sexually dimorphic characteristics of spinal microglia under neuropathic pain conditions are influenced by circulating androgens [9]. Further studies are needed to determine whether androgens modulate CCL4 expression and to elucidate the transcriptional mechanisms responsible for this regulation.

Overall, we demonstrated that the upregulation of CCL4 in the SDH following peripheral nerve injury occurs exclusively in males. Importantly, CCL4 may serve as a marker for male-dominant microglia involved in neuropathic pain. Although pharmacological inhibition of the CCL4–CCR5 pathway with i.t. maraviroc showed sexually dimorphic alleviation of neuropathic pain in the PSL model, activation of CCR5 via i.t. administration of exogenous CCL4 induced allodynia in both sexes. This suggests that the induction of CCL4 in activated microglia within the SDH may represent a crucial mechanism underlying sex differences in microglia-driven neuropathic pain. Considering that this sexual dimorphism in microglia is androgen-dependent, elucidating the regulatory mechanisms of spinal microglia mediated by androgen signaling can provide deeper insights into sex differences in pain processing.

## Data Availability

The original contributions presented in this study are included in the article/Supplementary Materials. Further inquiries can be directed to the corresponding authors.

## Supplementary Materials

The following supporting information can be downloaded at: https://www.mdpi.com/article/10.3390/cells14070484/s1, Figure S1: Expression of canonical microglia genes in all cell type clusters; Figure S2: Expression of *Ccl4* in male-dominant subpopulation; Figure S3: Expression profiling of *Ccl4* in mice.

## Abbreviations

The following abbreviations are used in this manuscript:

CCL4	CC-chemokine ligand 4
CCR5	CC-chemokine receptor 5
CSF1	colony-stimulating factor 1
GSEA	gene set enrichment analysis
PSL	partial sciatic nerve ligation
RT-qPCR	reverse transcription-quantitative polymerase chain reaction
scRNA-seq	single-cell RNA sequencing
SDH	spinal dorsal horn
SNI	spared nerve injury
UMAP	uniform manifold approximation and projection
