# Supplementary material for: Male-Dominant Spinal Microglia Contribute to Neuropathic Pain by Producing CC-Chemokine Ligand 4 Following Peripheral Nerve Injury
Source: Cells. 2025 Mar 23;14(7):484. doi: 10.3390/cells14070484 (PMC11987877; doi:10.3390/cells14070484)
Supplement: Supplementary file 1 [file cells-14-00484-s001.zip › cells-3524269-supplementary.pdf]

**Supplementary figures**

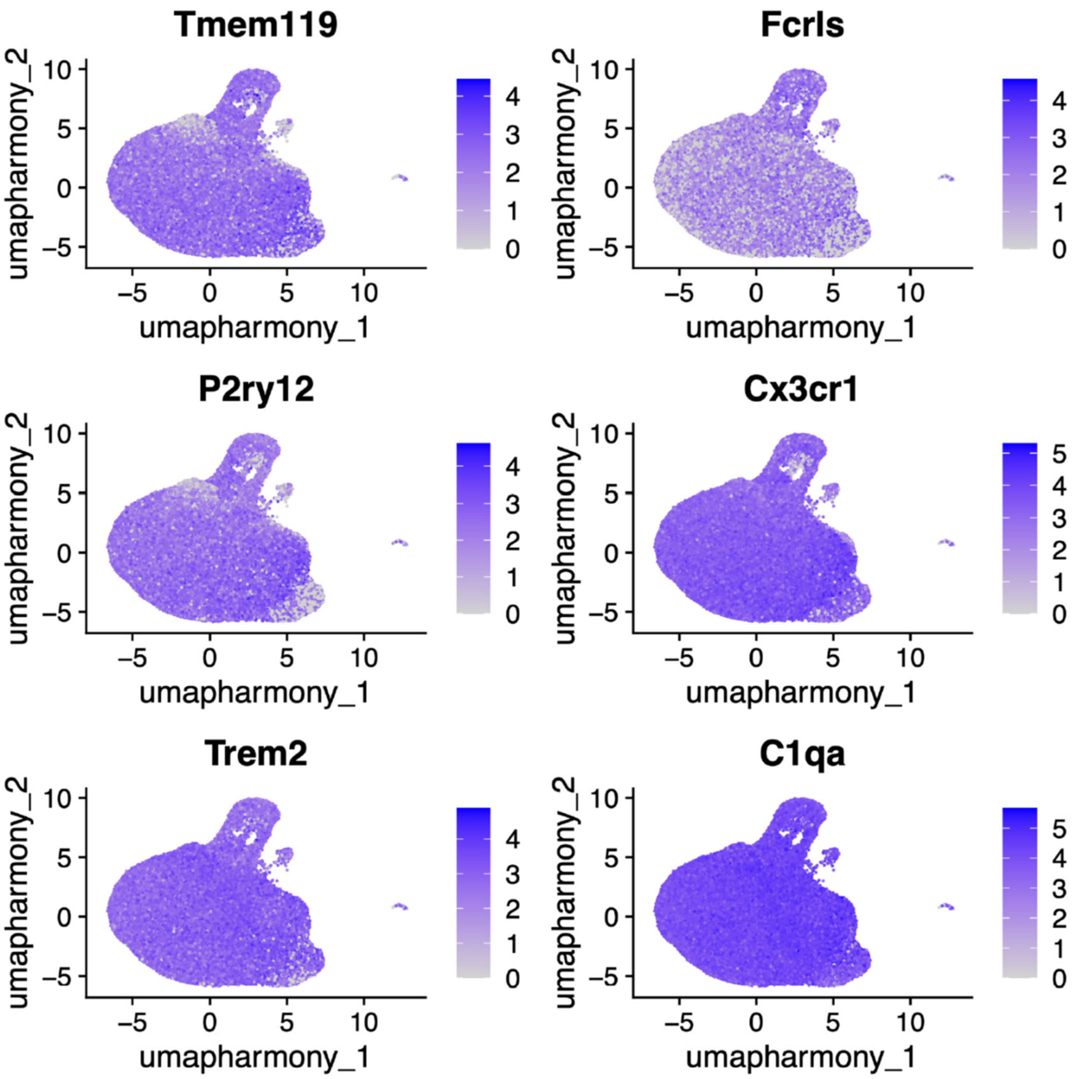


**Figure S1. Expression of canonical microglia genes in all cell type clusters.** *Tmem119*, *Fcrls*, *P2ry12*, *Cx3cr1*, *Trem2*, and *C1qa* were expressed in all 12 clusters shown in Figure 1.

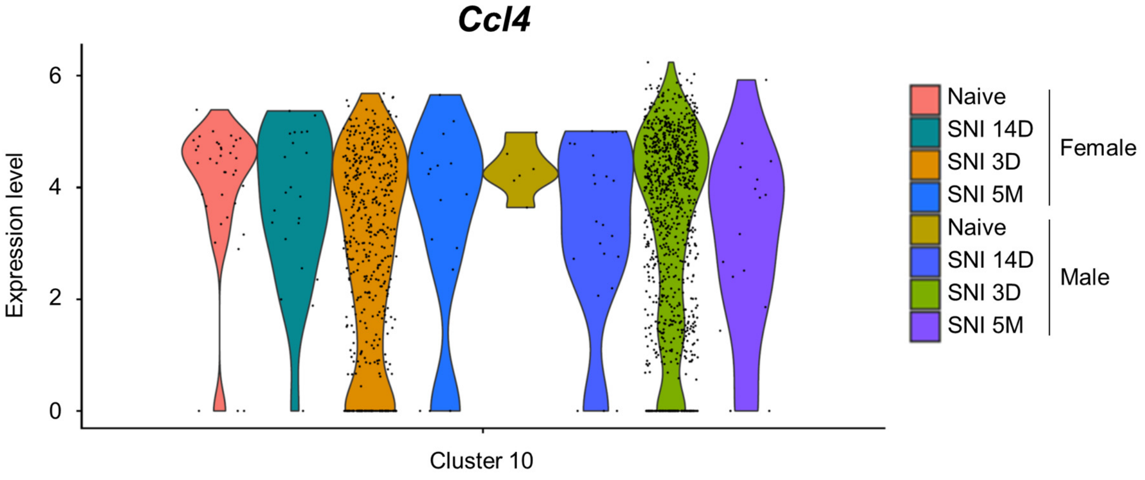


**Figure S2. Expression of *Ccl4* in male-dominant subpopulation.** Violin plot for *Ccl4* in cluster 10 microglia shown in Figure 1.

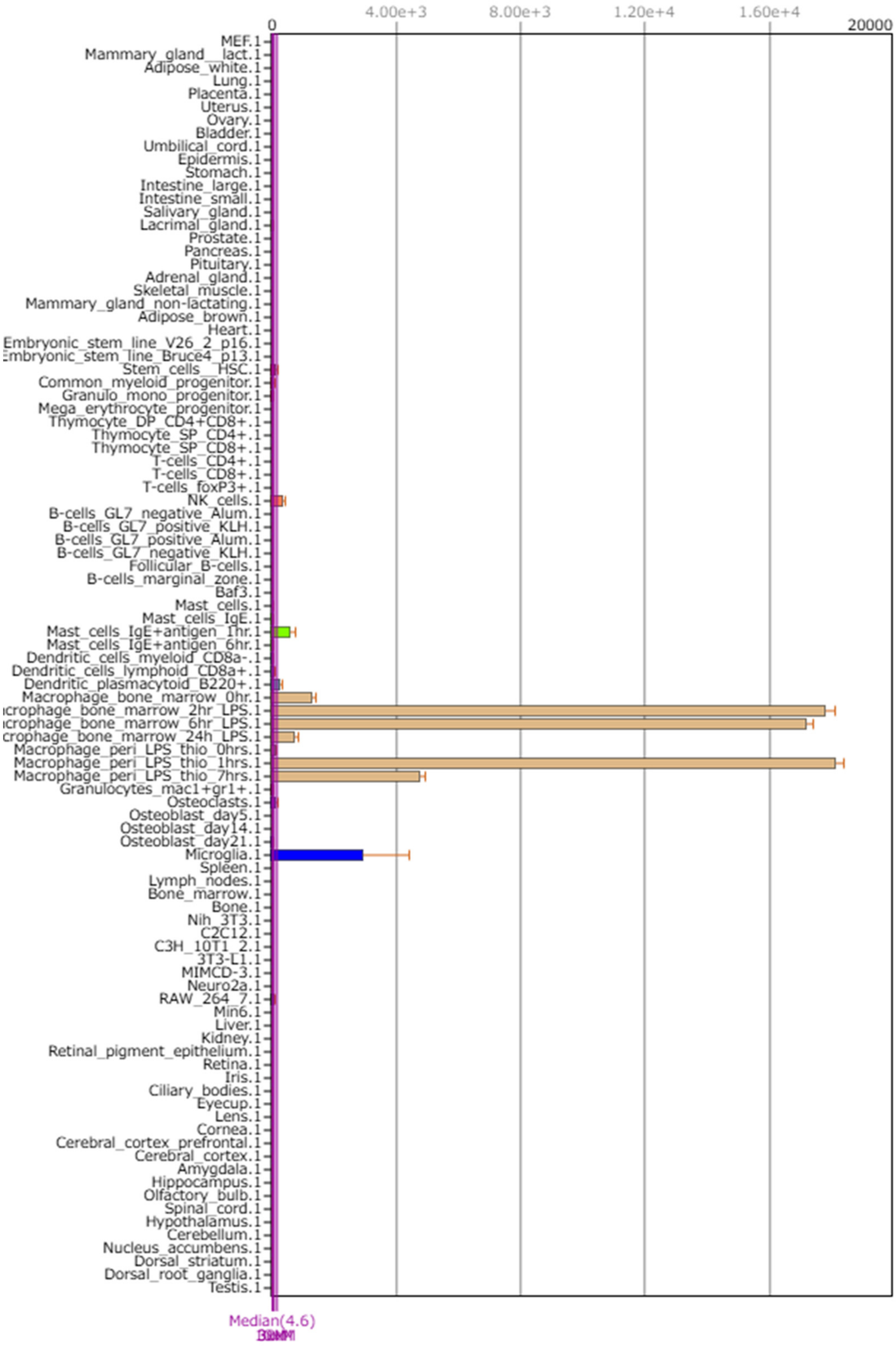


**Figure S3. Expression profiling of *Ccl4* in mice.** *Ccl4* expression across a diverse array GeneAtlas MOE430, gcrma. Probeset: 1421578_at.
